# Supplementary material for: A comparison of feature selection methodologies and learning algorithms in the development of a DNA methylation-based telomere length estimator
Source: BMC Bioinformatics. 2023 May 1;24:178. doi: 10.1186/s12859-023-05282-4 (PMC10152624; doi:10.1186/s12859-023-05282-4)
Supplement: Supplementary file 1 — Additional file 1. Performance metrics information, correlation plots, multiple linear regression results and blood cell count correlation tables. [file 12859_2023_5282_MOESM1_ESM.docx]

**A comparison of feature selection methodologies and learning algorithms in the development of a DNA methylation-based telomere length estimator**

**Supplementary Information.**

**Section 1:**

**Performance Metrics**

A range of performance metrics which are commonly used in DNA methylation-based regression studies (such as age estimation) are reported in this study. These include the Mean Absolute Error, the Mean Absolute Percentage Error (MAPE), Root Mean Squared Error (RMSE) and Pearson's correlation coefficient (r).

The Mean Absolute Error (MAE), Mean Absolute Percentage Error (MAPE), and Pearson's correlation coefficient (r_xy_) can be represented as:

$$\begin{aligned} MAE= n^{-1}\sum_{i=1}^{n} \left| x_{i}-x_{i}' \right|\#\left( 1 \right) \end{aligned}$$

$$\begin{aligned} MAPE= \frac{100}{n}\sum_{i=1}^{n} \left| \frac{x_{i}-{x_{i}}^{'}}{x_{i}} \right|\#\left( 2 \right) \end{aligned}$$

$$\begin{aligned} r_{xy}=\frac{n\sum X_{i}Y_{i}-\sum X_{i}\sum Y_{i}}{\sqrt{n\sum X_{i}^{2}-({\sum X_{i})}^{2}}\sqrt{n\sum Y_{i}^{2}-({\sum Y_{i})}^{2}}}\#\left( 3 \right) \end{aligned}$$

where *x_i_* and *x_i_'* are the actual and model predicted values for sample *i* respectively, *n* is the number of samples, *X_i_* and *Y_i_* are a pair of random variables [1].

**Supplementary Figures.**


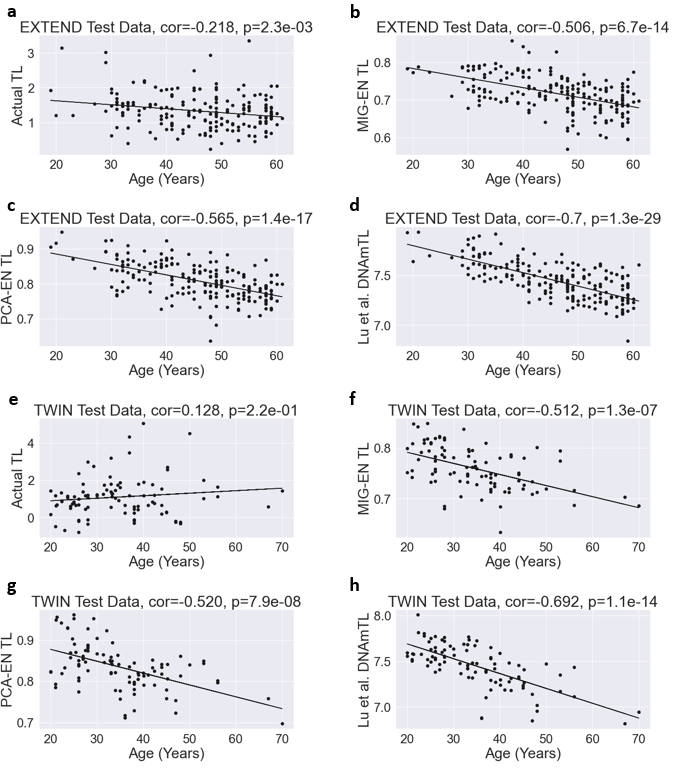


Figure S1: **a**. Chronological age vs. measured TL (EXTEND), **b**. Chronological age vs. MI-EN TL (EXTEND) and **C**. Chronological age vs. PCA-EN TL (EXTEND). **d**. Chronological age vs. measured TL (TWIN), **e**. Chronological age vs. MI-EN TL (TWIN), **f**. Chronological age vs. PCA-EN TL (TWIN), **g**. Chronological age vs. Lu et al. DNAmTL (EXTEND), **h**. Chronological age vs. Lu et al. DNAmTL (TWIN). Pearson's correlation coefficient and correlation test p-value is reported in each case. Samples sizes were n=192 for EXTEND and n=94 for TWIN data (age was not available for all twin pairs).


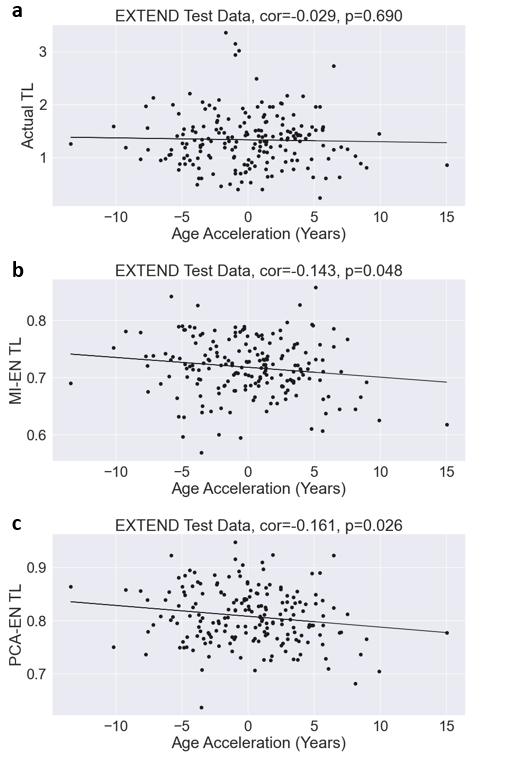


Figure S2: The x-axis variable is Age Acceleration, which represents the residual of the Lu et al. estimator DNAmAge [2] regressed on age. **A.** Age Acceleration vs. measured TL, **B.** Age Acceleration vs. MI-EN TL and **C**. Age Acceleration vs. PCA-EN TL. Plots relate to the EXTEND test data set and Pearson's correlation coefficient and correlation test p-value is reported for each case.


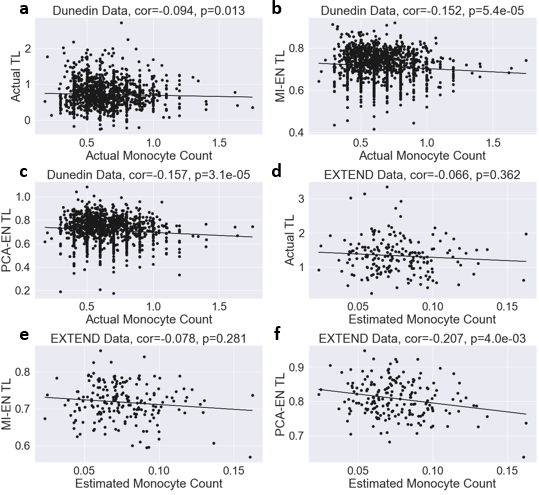


Figure S3: **A.** Actual Monocyte Count vs. measured TL, **B.** Actual Monocyte Count vs. MI-EN TL, **C**. Actual Monocyte Count vs. PCA-EN TL, **D.** Estimated Monocyte Count vs. measured TL, **E.** Estimated Monocyte Count vs. MI-EN TL, **F**. Estimated Monocyte Count vs. PCA-EN TL Dunedin data contained actual monocyte counts, EXTEND data set contained estimated monocyte counts. Pearson's correlation coefficient and correlation test p-value is reported for EXTEND data while repeated measures correlation was used for Dunedin data due to many donors having multiple samples.

**Supplementary Tables.**

Table S1: Multiple linear regression for analysis of biological correlates in the EXTEND data set. The top panel contains results from a multiple linear regression model analysis of the z-score transformed actual TL (dependent variable) on a range of covariates for the EXTEND data set (n=192). The model was regressed on age, sex, current smoking status and body mass index (BMI). The second, third and fourth panels contain results of analogous multiple regression models but with dependent variables of MI-EN TL, PCA-EN TL and DNAmTL (Lu et al. [3]) respectively. SE denotes the standard error.

| **Actual TL values** | | | |
| --- | --- | --- | --- |
| **Variable** | **Coefficient (SE)** | **t-statistic** | **P-value** |
| Intercept | 0.624 (0.491) | 1.27 | 0.205 |
| Age | -0.022 (0.007) | -3.03 | 2.83e-03 |
| Male | -0.329 (0.163) | -2.02 | 0.045 |
| Current Smoker | 0.006 (0.359) | 0.02 | 0.988 |
| BMI | 0.018 (0.015) | 1.20 | 0.232 |
| **MI-EN Predicted TL** | | | |
| Intercept | 0.813 (0.020) | 40.38 | 2.73e-94 |
| Age | -0.002 (0.000) | -8.07 | 8.49e-14 |
| Male | -0.035(0.007) | -5.28 | 3.61e-07 |
| Current Smoker | 0.015 (0.015) | 0.99 | 0.325 |
| BMI | 0.001 (0.001) | 1.46 | 0.145 |
| **PCA-EN Predicted TL** | | | |
| Intercept | 0.949 (0.021) | 44.86 | 5.05e-102 |
| Age | -0.003 (0.000) | -9.20 | 6.91e-17 |
| Male | -0.023 (0.007) | -3.30 | 1.18e-03 |
| Current Smoker | -0.008 (0.015) | -0.50 | 0.619 |
| BMI | -5.87e-05 (0.001) | -0.09 | 0.929 |
| **DNAmTL Predicted TL (Lu et al.)** |  |  |  |
| Intercept | 8.070 (0.067) | 120.4 | 3.85e-179 |
| Age | -0.013 (0.001) | -13.37 | 4.64e-29 |
| Male | -0.077 (0.022) | -3.45 | 6.96e-04 |
| Current Smoker | -0.029 (0.049) | -0.59 | 0.559 |
| BMI | 4.22e-04 (0.002) | -0.20 | 0.840 |

Table S2: Pearson correlation coefficients and p-values for both age-adjusted actual TL and imputed blood cell counts (EXTEND data) and actual TL and imputed blood cell counts (TWIN data).

| **data** | **var** | **cell** | **Correlation** | **P** |
| --- | --- | --- | --- | --- |
| EXTEND | TLadjAge | CD8.naive | 0.120 | 9.77E-02 |
|  | TLadjAge | CD8pCD28nCD45RAn | -0.046 | 5.24E-01 |
|  | TLadjAge | Plasma blast | 0.033 | 6.53E-01 |
|  | TLadjAge | CD4T | -0.153 | 3.42E-02 |
|  | TLadjAge | NK | -0.186 | 9.73E-03 |
|  | TLadjAge | Mono | -0.066 | 3.62E-01 |
|  | TLadjAge | Gran | 0.205 | 4.28E-03 |
| TWIN | TL | CD8.naive | 0.169 | 2.43E-02 |
|  | TL | CD8pCD28nCD45RAn | -0.098 | 1.94E-01 |
|  | TL | Plasma blast | 0.056 | 4.60E-01 |
|  | TL | CD4T | 0.127 | 9.0E-02 |
|  | TL | NK | -0.027 | 7.18E-01 |
|  | TL | Mono | -0.033 | 6.62E-01 |
|  | TL | Gran | 0.023 | 7.59E-01 |

Table S3: Pearson correlation coefficients and p-values for both age-adjusted MIG-EN TL and imputed blood cell counts (EXTEND data) and MIG-EN TL and imputed blood cell counts (TWIN data).

| **Data** | **var** | **cell** | **Correlation** | **P** |
| --- | --- | --- | --- | --- |
| EXTEND | MI-ENadjAge | CD8.naive | 0.268 | 1.75E-04 |
|  | MI-ENadjAge | CD8pCD28nCD45RAn | -0.171 | 1.79E-02 |
|  | MI-ENadjAge | Plasma blast | -0.099 | 1.74E-01 |
|  | MI-ENadjAge | CD4T | 0.191 | 8.11E-03 |
|  | MI-ENadjAge | NK | -0.160 | 2.69E-02 |
|  | MI-ENadjAge | Mono | -0.078 | 2.81E-01 |
|  | MI-ENadjAge | Gran | -0.039 | 5.92E-01 |
| TWIN | MI-EN TL | CD8.naive | 0.456 | 1.65E-10 |
|  | MI-EN TL | CD8pCD28nCD45RAn | -0.398 | 3.66E-08 |
|  | MI-EN TL | Plasma blast | -0.275 | 2.08E-04 |
|  | MI-EN TL | CD4T | 0.384 | 1.26E-07 |
|  | MI-EN TL | NK | -0.048 | 5.24E-01 |
|  | MI-EN TL | Mono | -0.161 | 3.19E-02 |
|  | MI-EN TL | Gran | -0.246 | 9.23E-04 |

Table S4: Pearson correlation coefficients and p-values for both age-adjusted PCA-EN TL and imputed blood cell counts (EXTEND data) and PCA-EN TL and imputed blood cell counts (TWIN data).

| **data** | **var** | **cell** | **Correlation** | **P** |
| --- | --- | --- | --- | --- |
| EXTEND | PCA-ENadjAge | CD8.naive | 0.395 | 1.49E-08 |
|  | PCA-ENadjAge | CD8pCD28nCD45RAn | -0.160 | 2.63E-02 |
|  | PCA-ENadjAge | Plasma blast | 0.196 | 6.55E-03 |
|  | PCA-ENadjAge | CD4T | -0.011 | 8.85E-01 |
|  | PCA-ENadjAge | NK | -0.159 | 2.73E-02 |
|  | PCA-ENadjAge | Mono | -0.207 | 4.02E-03 |
|  | PCA-ENadjAge | Gran | 0.260 | 2.72E-04 |
| TWIN | PCA-EN TL | CD8.naive | 0.617 | 4.48E-20 |
|  | PCA-EN TL | CD8pCD28nCD45RAn | -0.451 | 2.77E-10 |
|  | PCA-EN TL | Plasma blast | -0.343 | 2.74E-06 |
|  | PCA-EN TL | CD4T | 0.456 | 1.6E-10 |
|  | PCA-EN TL | NK | -0.001 | 9.90E-01 |
|  | PCA-EN TL | Mono | -0.123 | 1.03E-01 |
|  | PCA-EN TL | Gran | -0.278 | 1.97E-04 |

Table S5: Pearson correlation coefficients and p-values for both age-adjusted DNAmTL and imputed blood cell counts (EXTEND data) and DNAmTL and imputed blood cell counts (TWIN data).

| **data** | **var** | **cell** | **Correlation** | **P** |
| --- | --- | --- | --- | --- |
| EXTEND | DNAmTLadjAge | CD8.naive | 0.465 | 1.19E-11 |
|  | DNAmTLadjAge | CD4.naive | 0.313 | 9.60E-06 |
|  | DNAmTLadjAge | CD8pCD28nCD45RAn | -0.213 | 3.05E-03 |
|  | DNAmTLadjAge | Plasma blast | -0.093 | 1.97E-01 |
|  | DNAmTLadjAge | CD4T | 0.314 | 9.44E-06 |
|  | DNAmTLadjAge | NK | -0.170 | 1.88E-02 |
|  | DNAmTLadjAge | Mono | -0.214 | 2.82E-03 |
|  | DNAmTLadjAge | Gran | -0.055 | 4.47E-01 |
| TWIN | DNAmTL | CD8.naive | 0.685 | 5.47E-26 |
|  | DNAmTL | CD8pCD28nCD45RAn | -0.523 | 6.65E-14 |
|  | DNAmTL | Plasma blast | -0.4 | 3.21E-08 |
|  | DNAmTL | CD4T | 0.457 | 1.44E-10 |
|  | DNAmTL | NK | -0.013 | 8.67E-01 |
|  | DNAmTL | Mono | -0.062 | 4.09E-01 |
|  | DNAmTL | Gran | -0.368 | 4.22E-07 |

Table S6: Repeated measures correlation between actual TL/MI-EN TL/PCA-EN TL and actual blood cell counts.

| **data** | **var** | **cell** | **Correlation** | **P** |
| --- | --- | --- | --- | --- |
| Dunedin | Actual TL | Neutrophils | -0.035 | 0.34082800 |
| Dunedin | Actual TL | Lymphocytes | -0.036 | 0.33419100 |
| Dunedin | Actual TL | Monocytes | -0.094 | 0.01263800 |
| Dunedin | Actual TL | Eosinophils | -0.150 | 0.00009300 |
| Dunedin | Actual TL | Basophils | -0.242 | 0.01359000 |
| Dunedin | MI-EN TL | Neutrophils | -0.054 | 0.14370800 |
| Dunedin | MI-EN TL | Lymphocytes | -0.135 | 0.00026500 |
| Dunedin | MI-EN TL | Monocytes | -0.152 | 0.00005400 |
| Dunedin | MI-EN TL | Eosinophils | -0.096 | 0.01210500 |
| Dunedin | MI-EN TL | Basophils | -0.408 | 0.00001800 |
| Dunedin | PCA-EN TL | Neutrophils | -0.021 | 0.57911400 |
| Dunedin | PCA-EN TL | Lymphocytes | -0.134 | 0.00029700 |
| Dunedin | PCA-EN TL | Monocytes | -0.157 | 0.00003100 |
| Dunedin | PCA-EN TL | Eosinophils | -0.194 | 0.00000036 |
| Dunedin | PCA-EN TL | Basophils | -0.382 | 0.00006700 |

**References**

1. Willmott, C.J. and K. Matsuura, *Advantages of the mean absolute error (MAE) over the root mean square error (RMSE) in assessing average model performance.* Climate research, 2005. **30**(1): p. 79-82.

2. Horvath, S., *DNA methylation age of human tissues and cell types.* Genome biology, 2013. **14**(10): p. 3156.

3. Lu, A.T., et al., *DNA methylation-based estimator of telomere length.* Aging (Albany NY), 2019. **11**(16): p. 5895.
